# A Drop of Blood to Lead the Way

**DOI:** 10.3390/hematolrep17040040

**Published:** 2025-08-05

**Authors:** Theodora A. M. Claushuis, Marielle J. Wondergem, Henriette B. Beverloo, Marise R. Heerma van Voss, Remco J. Molenaar, Maud Zwolsman, Fleur M. van der Valk, Hans L. Mooij, Lianne Koens, Sanne H. Tonino

**Affiliations:** 1 Department of Hematology, Amsterdam University Medical Center, De Boelelaan 1117, 1081 HV Amsterdam, The Netherlands; m.j.wondergem@amsterdamumc.nl (M.J.W.); m.r.heermavanvoss@amsterdamumc.nl (M.R.H.v.V.); r.j.molenaar@amsterdamumc.nl (R.J.M.); s.h.tonino@amsterdamumc.nl (S.H.T.); 2 Department of Clinical Genetics, Erasmus Medical Center, Dr. Molewaterplein 40, P.O. Box 2040, 3000 CA Rotterdam, The Netherlands; h.b.beverloo@erasmusmc.nl; 3 Department of Internal Medicine, OLVG Hospital, Oosterpark 9, 1091 AC Amsterdam, The Netherlands; m.zwolsman@olvg.nl (M.Z.); f.m.vandervalk@olvg.nl (F.M.v.d.V.); h.l.mooij@olvg.nl (H.L.M.); 4 Department of Pathology, Amsterdam University Medical Center, De Boelelaan 1117, 1081 HV Amsterdam, The Netherlands; l.koens@amsterdamumc.nl

**Keywords:** hemophagocytic lymphohistiocytosis, Epstein–Barr virus, liquid biopsy, lymphoma

## Abstract

**Background and Significances:** In patients with Epstein–Barr virus-driven hemophagocytic lymphohistiocytosis (EBV-HLH), identifying the underlying cause poses a significant diagnostic challenge. HLH may precede overt disease, and early directed treatment for HLH can obscure histopathological findings. A liquid biopsy enables the detection of tumor-derived DNA from various sources, including cell-free DNA, circulating tumor cells, extracellular vesicles, and tumor-educated platelets, and might aid in this setting. **Case Presentation:** This case presents a young patient with EBV-HLH, in which genomic analysis of tumor-derived DNA from circulating tumor cells led to the diagnosis of an EBV-positive NK/T-cell lymphoma—where conventional tissue biopsies had failed. **Conclusions:** This report underscores the potential of the liquid biopsy as a valuable diagnostic tool in complex cases of EBV-HLH.

## 1. Detailed Case Description

A 32-year-old male presented with fever. His medical history consisted of a benign neoplasm of the thymus. He reported daily fevers up to 39° for the last three weeks, as well as fatigue, night sweats, and weight loss. He had been treated empirically with amoxicillin and azithromycin (due to his partner’s chlamydial infection) without improvement. Born in Spain, he had recently visited there but lived in the Netherlands for 8 years. He reported no animal exposure, insect bites, drug use, new medications, or unprotected sexual contact. The family history was unremarkable. On examination, he appeared moderately ill: temperature 38.6 °C, heart rate 100 bpm, blood pressure 110/70 mmHg, and oxygen saturation 95%. Mild hepatosplenomegaly was present.

**Figure 1:**
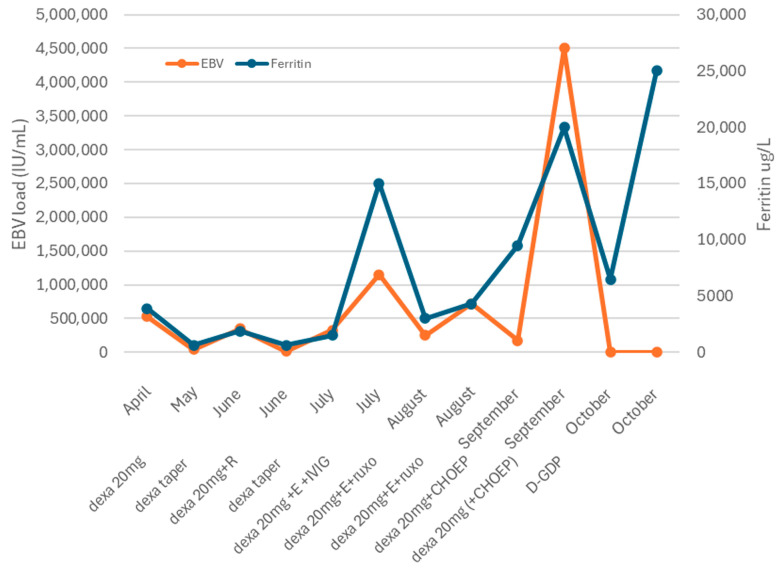
Clinical course. Abbreviations: Dexa = dexamethasone. R = rituximab. E = etoposide. IVIG: intravenous immunoglobulin. CHOEP: cyclophosphamide, doxorubicin, vincristine, and etoposide. D-GDP = cisplatin, dexamethasone, gemcitabine, and peg-asparaginase.

Initial laboratory analysis revealed a mild anemia (hemoglobin 8.0 mmol/L (reference range, 8–11.2), thrombocytopenia (74 × 10^9^/L (150–400)), and leukopenia 2.6 × 10^9^/L (4–10), with neutrophils of 1.0 × 10^9^/L (1.5–7.5), bilirubin of 6.3 µmol/L (<21), lactate dehydrogenase (LDH) 748 units/L (<233), haptoglobin < 0.1 g/L (0.5–2.2), and creatinine 83 µmol/L (60–110). The direct antiglobulin test was negative, and the peripheral-blood smear did not reveal schistocytes. Aspartate aminotransferase (AST) and alanine aminotransferase (ALT) were both increased (231 and 316 IU/L (8–35)) as along with gamma-glutamyl transpeptidase (G-GT, 123 IU/L (5–40)) and ferritin (3901 µg/L (20–330)). Coagulation tests revealed a normal international normalized ratio (INR) 1.0 (0.8–1.1), activated partial thromboplastin time of 31 s (30–40), and a low fibrinogen of 1.6 g/L (2.0–4.0) (Figure 1).

The combination of prolonged fever, hepatosplenomegaly, pancytopenia, hyperferritinemia, elevated liver enzymes, and hypofibrinogenemia suggests hemophagocytic lymphohistiocytosis (HLH).

The HScore at this time was 189, and there was a positive optimized HLH inflammatory index (OHI).

**Figure 2:**
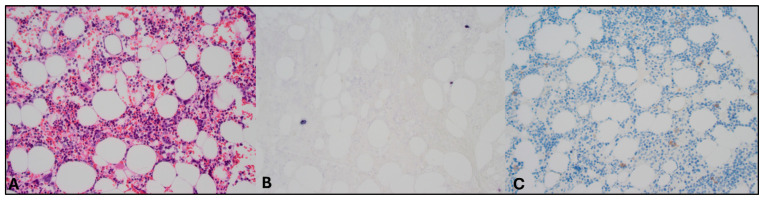
Pathology assessment of bone marrow biopsy specimens. Magnification 200×. Images evaluated with Olympus BX50 microscope (Olympus, Tokyo, Japan) and images scanned with a Pannoramic 480 scanner (3D Histech, Budapest, Hungary). (**A**) Hematoxylin–eosin staining revealed no abnormalities. (**B**) EBV and CD20 double staining showed sporadic EBV-positive (non-B) cells, but these cells could not be characterized further. (**C**) CD56 staining in this context of sporadic EBV-positive cells was difficult to interpret but did not identify the EBV+ cells. (Double) staining of other markers also did not identify the EBV positive cells (CD79a, PAX5, CD2, CD3, CD30, and granzyme B).

**Figure 3:**
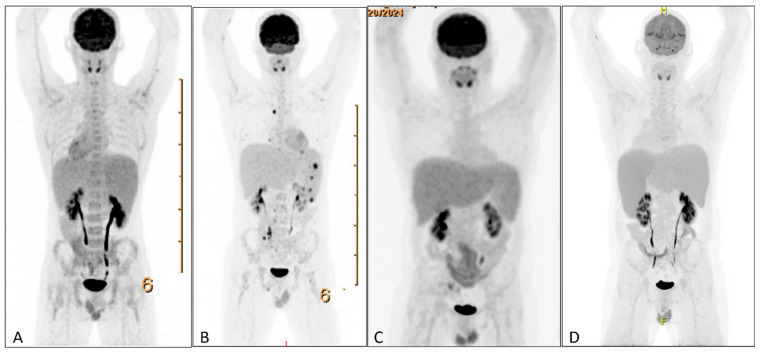
PET scans during clinical course. (**A**) PET-CT showing splenomegaly without increased FDG-uptake. (**B**) PET-CT showing additional mesenterial lymphadenopathy, FDG-avid splenic nodules, and bone lesions. (**C**) PET-CT showing normalization of FDG-avid lymphadenopathy and splenomegaly without increased FDG uptake. (**D**) PET-CT showing increased splenomegaly with increased FDG uptake. PET = positron emission tomography. FDG = ^18^fluorodeoxyglucose.

HLH can arise secondary to infections, autoimmune disorders, and malignancies, particularly lymphomas. Therefore, a broad diagnostic workup was initiated. Infectious screening revealed negative blood cultures, no malaria, and negative serologies for CMV; HIV; HSV-1/2; HHV-6/8; *Tropheryma whipplei*; Mycobacterium tuberculosis; hepatitis A, B, and C; *Treponema pallidum*; *Toxoplasma gondii*; and *Leishmania* spp. PCR testing revealed EBV DNA at 533,000 copies/mL, with positive EBNA IgG and VCA IgG and negative IgM. Urine tests were negative for *M. tuberculosis*, *Histoplasma* spp., Chlamydia trachomatis, and Neisseria gonorrhoeae. Hematologic evaluation showed no clonal B- or T-cell populations on flow cytometry or clonal T-cell receptor gene rearrangements on peripheral blood. Autoimmune serologies (ANA and anti-dsDNA) were negative. Also the bone marrow biopsy showed no morphological or immunophenotypic evidence of malignancy, infection, or clonal lymphocytes. Stains for microbial agents were negative, and the karyotype was normal. Scattered EBV-positive cells were present but too sparse for further characterization (Figure 2A–C). A PET scan revealed splenomegaly (18 cm, Figure 3A) without significant FDG uptake, increased bone marrow uptake, and no thymic abnormalities. The patient was admitted for further workup, and dexamethasone 20 mg daily was initiated.

This 32-year-old man presented with HLH and high EBV load. At this point, there are several etiologies to be considered. Although primary EBV infection can induce HLH [1], the positive EBV IgG suggested this was not the case. EBV reactivation in primary immunodeficiencies—such as interleukin-2-inducible t-cell kinase (ITK) deficiency, autoimmune lymphoproliferative syndrome (ALPS), and X-linked lymphoproliferative disease (XLP) [2,3]—remains possible, given their association with EBV-related HLH. Chronic active EBV infection (CAEBV), defined by persistent EBV viremia > 3 months, organ involvement, and EBV-positive T or NK cells without known immunodeficiency or malignancy [4] and also EBV-positive lymphoma (NK/T-cell, B-cell, or Hodgkin lymphoma) should also be considered. Aggressive NK-cell leukemia, typically EBV-associated, seemed unlikely as the bone marrow showed no malignant infiltration. Finally, EBV might act as a non-specific HLH trigger, either in familial HLH or critical illness.

**Figure 4:**
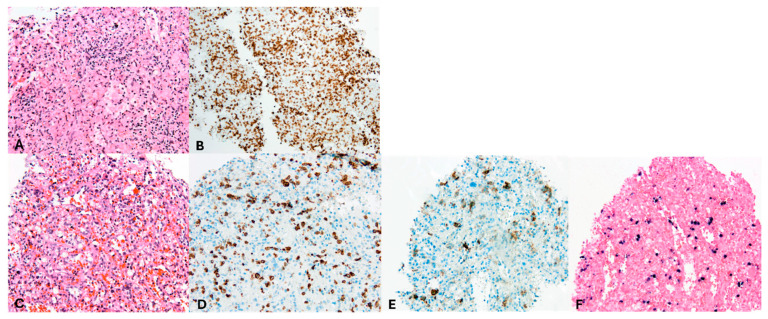
Pathology assessment of spleen biopsies. Magnification 200×. Images evaluated with Olympus BX50 microscope and images scanned with a Pannoramic 480 scanner (3D Histech). (**A**,**B**) second spleen biopsy, (**C**–**F**) third spleen biopsy. (**A**) Hematoxylin–eosin and (**B**) CD3 staining revealed a diffuse infiltrate of small T cells without evident abnormalities in the second spleen biopsy; however, limited material precluded any further staining or clonality assessment. (**C**) Hematoxylin–eosin, (**D**) CD3, (**E**) CD56, and (**F**) EBV staining of the third spleen biopsy showing numerous large, scattered, atypical cells consistent with an EBV-positive extranodal NK-/T-cell lymphoma.

Following dexamethasone, the patient improved, allowing discharge (Figure 1). A splenic biopsy showed active inflammation with EBV-encoded RNA (EBER) positivity but no evidence of B- or-T-cell lymphoma, including assessment of clonality. As this case involved an outpatient, ferritin and EBV load decreased, and dexamethasone was tapered. Whole-exome sequencing (WES) for primary immunodeficiency and HLH-related genes was negative. Six weeks later, fever recurred with new FDG-avid splenic nodules, bone lesions, and mesenteric lymphadenopathy on PET-CT (Figure 3B). Mesenteric biopsies were inconclusive; repeating the splenic biopsy showed no lymphoma but a reactive T-cell influx, though limited tissue precluded further analyses (Figure 4A–C). Skin biopsies showed no intravascular lymphoma. Re-treatment with dexamethasone and rituximab transiently reduced EBV load and ferritin. However, upon another rise in ferritin and EBV levels, HLH-94 therapy (dexamethasone, etoposide, and IVIG) was initiated, without lasting response. Ruxolitinib was added, achieving only temporary improvement, and the patient was transferred to the academic hospital (Figure 1).

At this point, the patient has been treated with multiple lines of therapy for an EBV-related HLH. Genetic analysis did not reveal primary immune deficiencies or genes related to familial HLH, though these cannot be fully excluded. Extensive biopsies failed to show lymphoma, though interpretation was limited by concurrent steroid use. CAEBV remains a possibility, even though the EBV serology was not typical for CAEBV and infectious mononucleosis-like symptoms were not clearly present in the 3 months before presentation. Additional cell sorting to detect EBV-infected B, NK, and T cells could support this diagnosis.

After referral, PET-CT was repeated and showed resolution of prior lymphadenopathy and no splenic FDG uptake, though splenomegaly persisted (18 cm; Figure 3C). Cell sorting revealed high EBV loads in NK cells (500,000 copies/10^5^ cells), T cells (90,000), and monocytes (200,000), supporting CAEBV. Additional analyses for AP/XIAP expression and NKG2D were negative. With the working hypothesis of CAEBV, CHOP-etoposide was initiated, and allogeneic stem cell transplantation was planned, but an EBV flare recurred within two weeks. At this point, peripheral blood sent for the immune deficiency panel was analyzed using a WES-based panel for the presence of DNA structural variants and DNA copy number abnormalities. Due to an aberrant pattern for the DNA copy number analysis, which could not be of constitutional origin, additional whole-genome SNP-array analysis was performed. In 20–50% of cells this resulted in (XY, +X[0.2], del(X)(q11q26)[0.2], dup(2)(q23q35)[0.3], +6[0.2], dup(6)(q12q16)[0.3], del(6)(q16)[0.2], del(6)(q16q25)[0.2], hmz(17)(pterp11)[0.5], +20[0.3], del(20)(p12)[0.3]). These copy number changes might be consistent with a malignancy of lymphoid origin. In light of these cytogenetic abnormalities, specifically, loss of heterozygosity of a large part of the short arm of chromosome 17, whole-exome sequencing data on peripheral blood was re-evaluated, showing a pathogenic variant in TP53 on 17p13 (NM_000546.6(TP53):c.817C>T, p.(Arg273Cys)), with a variant allele frequency of 31%.

The detection of a pathogenic TP53 variant in peripheral blood combined with lymphoid cytogenetic abnormalities and EBV-positive T and NK cells strongly indicated an underlying EBV-positive NK/T-cell lymphoma. SNP-array karyotyping and NGS of peripheral blood was pivotal in establishing this diagnosis when biopsies could not.

During a subsequent HLH flare, PET-CT revealed progressive splenomegaly with increased FDG uptake (Figure 3D). A third splenic biopsy showed an atypical infiltrate of small and scattered larger cells with irregular nuclei. The cells were positive for CD2, CD3, CD56, granzyme B, TIA1, and EBER; CD56/EBER double staining confirmed the atypical population. CD30 was weakly positive (1–5%). p53 showed nuclear overexpression with high proliferative activity (75%). CD5, CD7, CD8, MUM1, TCRab, TCR-delta, TdT, and CD20 were negative. No clonal T-cell population was detected. The findings support EBV-positive extranodal NK/T-cell lymphoma (Figure 4C–F). The patient was treated with D-GDP (cisplatin, dexamethasone, gemcitabine, and peg-asparaginase) [5], resulting in clinical improvement and a reduction in EBV load and ferritin levels. Ruxolitinib and dexamethasone were tapered.

However, the patient developed severe infectious complications, including Gram-negative bacteremia and pulmonary aspergillosis, followed by hyperbilirubinemia and hepatotoxicity, likely related to peg-asparaginase. Additionally, encephalopathy of unclear etiology developed, with no evidence of infection, CNS lymphoma, HLH activity, or liver failure. Despite aggressive management, the patient progressed to multi-organ failure and ultimately succumbed to his disease.

## 2. Materials and Methods

Several methods were used in the diagnostic process of this patient. EBV on pathology slides was detected by EBER in situ hybridization (Ventana). EBV load was detected by PCR on isolated DNA by detecting and amplifying BNRF1 (fragment of EBV nonglycosylated membrane protein), compared to an external standard, by using the LightCycler 480 (Roche Applied Sciences, Penzberg, Germany).

Cell sorting was performed on at least 5 × 10^6^ Peripheral Blood Mononuclear Cells (PBMCs) isolated from blood. PBMCs were mixed with several fluorochrome labeled antibodies: a-CD16, a-CD56, a-CD14, a-CD22, and a-CD3 (Biolegend, San Diego, CA, USA or BD Biosciences, Milpitas, CA, USA). After incubation, cells were washed and sorted by using a FACSAria FACSsorter (BD Biosciences) by sorting CD3+CD16/56-cells as T cells, CD3-CD16/56+ cells as NK cells, and CD14+ cells as monocytes. Next, sorting purity was determined by flow cytometry, and cell fractions were mixed with lysis buffer and DNA extracted using MagNA Pure 96 (Roche Applied Sciences). EBV PCR was performed as described above. EBV load is quantified as IU EBV per 10^5^ cells.

Genetic analysis was performed as follows: DNA was hybridized onto an Illumina Infinium GSA+MD-24 v3.0, which after scanning was analyzed using NexusClinical analysis software (Bionano, San Diego, CA, USA).

The whole genome was analyzed with a general resolution of approximately 150 kb for the presence of copy number variations (UCSC Genome Browser March 2009 (NCBI37/hg19)). DNA was enriched using the Agilent SureSelectXT Human All Exon V7 capture kit (Agilent, Santa Clara, CA, USA) and paired-end sequenced on the Illumina platform (outsourced). Sequencing data were demultiplexed with bcl2fastq2 Conversion Software from Illumina (San Diego, CA, USA). The Illumina DRAGEN Bio-IT Platform was used for read mapping to the hg19 genome and sequence variant detection. Sequence variants and copy number variants were restricted to the genes present in the Primary Immunodeficiency Panel v14 (for content, see https://www.erasmusmc.nl/genoomdiagnostiek accessed date 1 July 2024; select Genoomdiagnostiek DNA and Primary Immunodeficiency Panel info file v14). The detected sequence variants were annotated and filtered with Alissa Interpret software and classified with Alamut Visual. Copy number variant detection was performed using the BAM multiscale reference method using depth of coverage analysis and dynamical bins in NexusClinical. The detected copy number variants were annotated and filtered with the NexusClinical software and classified using UCSC Genome Browser (NCBI37/hg19). Re-evaluation of the BAM file for the SNV 17p13 (NM_000546.6(TP53):c.817C>T, p.(Arg273Cys)), showed this variant to be present with a variant allele frequency of 31%.

## 3. Discussion

Diagnosing the underlying cause of (EBV-)HLH remains a significant clinical challenge due to its broad differential diagnosis [6] and the need for a comprehensive and often invasive diagnostic workup. HLH may precede overt manifestations of an underlying disease, and early initiation of immunosuppressive or cytotoxic therapy—often necessary to control life-threatening inflammation—can obscure histopathological features and hamper immunophenotyping. In addition, tissue biopsies may be inconclusive, either due to sampling limitations or inadequate tissue quality, particularly when inaccessible organs such as the spleen are involved.

In cases of HLH with no identifiable underlying cause, splenectomy can also provide diagnostic insight. Studies have demonstrated that histopathological examination of splenic tissue can uncover otherwise undetectable (cryptic) lymphomas, allowing for appropriate and timely treatment [7].

However, splenectomy and other invasive tissue biopsies are not without risk. These procedures may be contraindicated in critically ill patients, and delays associated with obtaining tissue can be detrimental in the rapidly progressing course of HLH. These limitations highlight the growing need for alternative, minimally invasive diagnostic modalities.

The liquid biopsy offers a non-invasive alternative, detecting tumor-derived DNA. It is often performed on cell-free DNA from plasma (circulating tumor (Ct)DNA) but can also use circulating tumor cells, vesicles, or tumor-educated platelets.

In our patient, genomic analysis of tumor-derived DNA from circulating tumor cells was used.

Techniques like NGS, SNP-based karyotyping, and immunoglobulin heavy chain rearrangement analyses show high sensitivity and specificity in detecting lymphomas like diffuse large B cell lymphoma (DLBCL) and intravascular lymphoma [8,9,10,11]. In DLBCL, a liquid biopsy based on lymphoma-related genetic alterations detected in ctDNA can reveal disease despite negative imaging [12,13] and is increasingly used to assess minimal residual disease. Its ability to detect clinically covert disease is also illustrated by the NIPT (prenatal testing, a whole-genome sequencing test) where genetic abnormalities in peripheral blood can precede other features of a malignant disease in asymptomatic women [14], demonstrating that the liquid biopsy is a sensitive and powerful tool. Moreover, the liquid biopsy can capture genomic information from multiple tumor sites simultaneously by detecting diverse cancer-derived components circulating in the blood. Since different anatomical locations may harbor distinct genetic mutations or chromosomal abnormalities, this multi-source detection can provide a more comprehensive overview of tumor heterogeneity and disease complexity than single-site tissue biopsies alone [11,15].

## 4. Future Research Directions and Possible Applications

Though not yet validated in HLH, a liquid biopsy could facilitate earlier lymphoma diagnosis when conventional methods fail.

To our knowledge, there are currently no publications on the use of the liquid biopsy in HLH. A study is ongoing to assess whether a ctDNA-based liquid biopsy in HLH can diagnose lymphoma earlier (NCT05702502). Its implementation may help the diagnostic process and enable earlier adequate treatment, specifically, in the absence of disease on imaging or in pathology samples.

## 5. Conclusions

In conclusion, this report shows how performing a liquid biopsy can aid in the search for underlying diagnosis in (EBV-driven) HLH.

## Data Availability

Data is unavailable due to privacy restrictions.
